# Animal welfare research is fascinating, ethical, and useful—but how can it be more rigorous?

**DOI:** 10.1186/s12915-023-01793-x

**Published:** 2023-12-29

**Authors:** Georgia J. Mason

**Affiliations:** Campbell Centre for the Study of Animal Welfare/Integrative Biology Department, University of Guelph, Guelph, Ontario N1G 2W1 Canada

## Abstract

**The scientific study of animal welfare supports evidence-based good animal care, its research contributing to guidelines and policies, helping to solve practical problems caused by animal stress, and raising fascinating questions about animal sentience and affective states. However, as for many branches of science (e.g. all those with replicability problems), the research rigour of welfare science could be improved. So, hoping to inspire methodologies with greater internal, external, and construct validity, here I outline 10 relevant papers and provide potential “journal club” discussion topics.**

## Welfare science now: a thriving field with ethical, practical, and fundamental relevance

As noted by Marian Dawkins, a long-standing leader in this field, animals with good welfare are healthy and have what they want (in terms of, for example, space, shelter, and opportunities to perform highly motivated natural behaviours). This results in them having more positive “affective states”, i.e. moods, emotions, and similar. Identifying such states, and understanding how they could be achieved, is the remit of animal welfare research. Studying animal welfare was somewhat fringe when the field emerged in the 1970s and 1980s: a European eccentricity. But today, animal welfare publications number in the thousands annually; animal welfare conferences involve hundreds of researchers; welfare presentations are not uncommon at agricultural, ecology, animal cognition, and even human emotion meetings; welfare research happens in BRICS and developing nations, not just the developed world; and in many countries, welfare research informs policies on how to treat animals. In parallel, welfare research techniques have become more sophisticated, often inspired by studies of human well-being (e.g. mood-sensitive cognitive changes like “judgment bias”).

The growth of welfare science partly reflects its ethical importance, along with increased acceptance by other branches of biology. It also reflects the rewarding nature of working in this field. Intellectually, welfare research touches on fascinating scientific questions such as the evolutionary functions of emotions and moods and the distribution of sentience. Furthermore, despite some tensions between human interests and animal needs (especially in agriculture), understanding and improving welfare can also help solve some practical problems: reducing behavioural problems in pets, tackling poor reproduction in zoos and conservation breeding centres, and increasing job satisfaction for laboratory animal technicians, to name a few. Welfare science is truly an absorbing, satisfying field to be in.

## Welfare science in the future: towards greater rigour and validity

*BMC Biology’s* twentieth anniversary collection comprises comment articles that provide an overview of different fields and projection of future trends, limited to referencing 10 papers. What to cover in my piece? The promise of new technologies for automated welfare assessment? How human research could reveal the functions of conscious affect? The need for wild animal welfare studies in a time of climate change? So many topics, yet underpinning all is a bedrock need for welfare science to be valid: to say something true and relevant about the animals it aims to understand. Validity is therefore my focus, especially given today’s understanding of the unintended consequences of academia’s “publish or perish” culture. I collate 10 papers and provide discussion topics (Table 1) for an imaginary journal club on internal, external, and construct validity. A perfect introduction is a seminar by Hanno Würbel, on the principles of good welfare science (https://www.youtube.com/watch?v=SXJ1TDEUf3U&t=1666s). Overall, I hope to provoke enjoyable debate, (perhaps uneasy) self-reflection, and ultimately more transparent, valid research.

**Table 1:** Discussion topics for each paper

**Paper**	**Discussion points**
**Internal validity: Are our studies bias-free and replicable?**	
Würbel (2017) [1] (other informative works include those by Leonard Freedman and Glenn Begley)	If only 50% biomedical experiments are replicable, what might the equivalent metric be for welfare research? If a welfare study proves non-replicable, does this matter most for the animals who were the subjects, those who are the “real-world” applied targets, policy makers, or future scientists? Würbel lists nine factors that can reduce replicability, often acting in different ways. How can underpowered studies do this, for instance? How can non-randomisation? And which might particularly impact the replicability of animal welfare research?
Kilkenny et al. (2009) [2] (see also https://arriveguidelines.org/arrive-guidelines. Other informative articles include ones by Dorothy Bishop, John Ioannidis, Malcolm Macleod, and Emily Sena)	Should the authors have considered blinding in all studies, not just those using subjective scoring? Do the findings surprise you? Which worries you most and why? If similar surveys were conducted of animal welfare research, what might they find?
Tuyttens et al. (2014) [3] (see also a 2016 follow-up study and interesting work by Nicole Nelson)	This work used veterinary students. Would biases be even stronger in people concerned about publication? In your research, are those who handle animals blind to hypothesis and/or treatment groups? Are those collecting the data? Are those analysing the data? What are the merits (e.g. in terms of practicality) of these different types of blinding?
Lazic (2010) [4]	If 12–48% of neuroscience papers may have pseudoreplicated, what might a similar survey of welfare research find? How does a legitimate repeated measures model differ from pseudoreplication? And if cage/pen is not the experimental unit to which a treatment is applied, do you still include it in your models?
**External validity: Are our studies relevant to real-world situations?**	
Dawkins 2012 [5]	Dawkins urges for more studies in commercial agricultural facilities. Do the benefits outweigh the costs? If you do not do this, what are the barriers? And are you worried that your data then lack generalisability to the populations needing them?
**Construct validity: Do our measures mean what we think they mean?**	
Rosso et al. (2022) [6]	This meta-analysis shows the dangers of indicators that can be interpreted in diverse ways, and HARK-ing’s seductive pull. Are the indicators you use, and the meanings of increased/decreased values, always clear before starting an experiment? Have you ever “spun” an effect to make it fit expectations? When is hypothesis-generation good but HARK-ing dangerous?
Mason & Veasey (2010) [7]	Do the three construct validation methods make sense, and are there are additional ones? Are the authors pessimistic or realistic, when they state “no one single welfare index is perfect”? Should a welfare indicator’s imperfections be factored into its use? For example, could it be useful to consider *a priori* the ‘false-negative’ and ‘false-positive’ results that an indicator is prone to, before use in welfare assessment?
Browning (2023) [8]	What do you think of conceptualising welfare as a “hidden target”? Do you like the way causes and effects of poor welfare are parsed out? What do you think of Browning’s recommended tests for robustness? And how often do you feel welfare research follows the logical pathways laid out here?
Sandem et al. (2002) [9] + three follow-up experiments using additional manipulations including a pharmacological treatment	Are you impressed at the range of situations used in this validatory research? Should eye white have been scored blind? Should future work check that arousal is not a confound? What other species might this eye white metric be useful in?
**To end**	
Muñoz-Tamayo et al. (2022) [10]	What do you think of this guide to open research? Are you comfortable sharing data (or organised enough to do so!)? Are you tempted by pre-registered reports? Should the journals used by welfare scientists change practices at all?

## Internal validity: are our studies bias-free and replicable?

Preclinical animal research (aiming to understand human disease) has been subject to devastating scrutiny especially around “spectacular cases of irreproducibility” [1]. Only half — at best — of biomedical studies are replicable, impeding biomedical progress with vast numbers of false leads. Causes include research designs that bias data (e.g. absence of blinding or randomisation), statistical misbehaviours like “P-hacking”, and selective reporting of results [1]. A survey of 271 biomedical publications thus identified “a number of issues” [2], randomisation being reported in just 12% for example. Practices like blinding are crucial in welfare research too, as Tuyttens and colleagues [3] demonstrated. Students, trained to extract data from ethological videos, produced skewed data if given false information about the subjects being scored (cattle believed to be hot being scored as panting more, for instance), leading the authors to lament, “can we believe what we score, if we score what we believe?”.

Adding further concerns, Kilkenny and colleagues found that only 62% of biomedical experiments that were amenable to factorial designs actually used them. Reassuringly, 87% did seem to use appropriate statistical methods [2]. However, P-hacking is often impossible to detect post-publication. Furthermore, other work (e.g. excellent publications by Stanley Lazic, including [4]) identifies pseudoreplication as a common statistical error. The Kilkenny paper also reported some lack of clarity in writing, inconsistent with *a priori* hypothesis testing, with 5% of studies not explaining their aims. (This issue resonated with me; in my lab, we recently screened the introductions of 71 papers on judgement bias and found it impossible to ascertain the research aims of 8 of these [11%]).

## External validity: are our studies relevant to real-world situations?

Even when results are internally valid and replicable, they might be irrelevant to other populations or contexts. Thus, biomedical research results often do not translate to humans; and for animal welfare, data collected in a welfare research lab may not translate to commercial situations. Solutions to this could include “introducing systematic variation (heterogenization) of relevant variables (for example species/strains of animals, housing conditions, tests)” [1]. Dawkins [5] takes this further, arguing that, at least for poultry, controlled laboratory situations have limited value. “Working directly with the poultry industry on commercial farms … shows what works in practice, out there in the real world”: it is critically important because “what is true of 50 birds in a small pen is not necessarily true of 50,000 birds in a large poultry house”.

## Construct validity: do our measures mean what we think they mean?

Welfare researchers have another challenge: making defensible inferences about something that cannot be measured directly — affective states. Doing this well requires knowing our measures have construct validity, and understanding *a priori* their strengths and weaknesses. Welfare studies thus largely fall into two types: those seeking to validate new indicators of affect (via manipulations known *a priori* to influence affective state) and those using well-validated indicators to discover new things about animal well-being. Both must be logical and transparent. Thus, validation studies must use defensible validation methods; and if a potential indicator fails, that measure must not be treated as if still valid. Likewise, welfare studies must select well-validated, appropriate indicators, such that increased/decreased values have meanings that are known *a priori*, not invoked *post hoc* once results are known.

If we do not work in this logical way, we risk “HARK-ing” (‘Hypothesising After the Results are Known’): a form of circular reasoning where aims and predictions are covertly tweaked after seeing patterns in the data, which looks (indeed *is*) biased. Perhaps worse, we may draw mistaken conclusions about animals: ones which fail to improve their well-being. As Rosso et al. [6] argue in a preprint, “HARKing can invalidate study outcomes and hamper evidence synthesis by inflating effect sizes... lead researchers into blind alleys … and waste animals, time, and resources”.

So, how to ensure an indicator has construct validity? Jake Veasey and I [7] outlined three methods: (1) assessing whether a potential indicator changes alongside self-reported affect in humans (assuming homology between species), (2) assessing whether it changes in animals deliberately exposed to aversive treatments, and (3) assessing whether such changes can be reversed pharmacologically, by giving, e.g. analgesics or anxiolytics. Another two — as beautifully laid out by philosopher Heather Browning [8] — are as follows: (4) recording effects of exposing animals to factors important for fitness and (5) identifying correlates of existing, well-validated indicators. And to give one illustration of construct validation done well, Agnethe-Irén Sandem and colleagues investigated eye-white exposure as a potential indicator of negative affect in cattle (e.g. [9]); see Table 1 for details.

## To end

Underneath all these issues lies the problematic incentive structure of academia. As Richard Horton, editor of *The Lancet*, wrote in 2015, “No-one is incentivised to be right. Instead, scientists are incentivised to be productive”. Obsessions with publication rates and *P*-values under 0.05 affect animal welfare science just as they do other disciplines. One partial solution could involve “open science” practices [10], such as pre-registering planned studies (so that hypotheses and statistical analyses are spelled out *a priori*, and, for registered reports, manuscripts are peer-reviewed and accepted before results are generated) and providing open access to data (so that anyone can re-analyse them). But perhaps more radically, perhaps a more fundamental overhaul is needed: a transition to a slower, better science that could improve researchers’ welfare as well as animals'?

## Data Availability

Not applicable.
